# Synthesis and Characterization of Zinc Oxide Nanoparticles Stabilized with Biopolymers for Application in Wound-Healing Mixed Gels

**DOI:** 10.3390/gels9010057

**Published:** 2023-01-11

**Authors:** Andrey V. Blinov, Maksim D. Kachanov, Alexey A. Gvozdenko, Andrey A. Nagdalian, Anastasiya A. Blinova, Zafar A. Rekhman, Alexey B. Golik, Dmitriy S. Vakalov, David G. Maglakelidze, Anzhela G. Nagapetova, Alexander D. Pokhilko, Irina V. Burkina

**Affiliations:** 1 Department of Physics and Technology of Nanostructures and Materials, Physical and Technical Faculty, North Caucasus Federal University, 355017 Stavropol, Russia; 2 Department of Medicine, Faculty of Therapy, Kuban State Medical University, 355000 Krasnodar, Russia; 3 Laboratory of Food and Industrial Biotechnology, Faculty of Food Engineering and Biotechnology, North Caucasus Federal University, 355017 Stavropol, Russia; 4 Armavir State Pedagogical University, 352901 Armavir, Russia; 5 Stavropol State Pedagogical University, 355017 Stavropol, Russia

**Keywords:** ZnO, sol–gel method, rheological properties, neural networks, multifactorial experiment

## Abstract

A method for the synthesis of ZnO nanoparticles (ZnO NPs) gels was developed. ZnO NPs were obtained through a sol–gel method with zinc acetate usage as a precursor. Optimization of the method of synthesis of ZnO NPs gel has been carried out. It was observed that the most stable ZnO NPs gels are formed at room temperature, pH = 8 and molar concentration of zinc C(Zn^2+^) = 0.05–0.2 M. It was shown that the addition of polysaccharide significantly affects the rheological properties and microstructure of ZnO NPs gels. We found that the optimal polysaccharide for the synthesis of ZnO NPs gels is hydroxyethyl cellulose. It is shown that the microstructure of a gel of ZnO NPs stabilized with hydroxyethyl cellulose is represented by irregularly shaped particles that are assembled into aggregates, with sizes ranging from 150 to 1400 nm. A significant hysteresis region is observed in a gel of ZnO NPs stabilized with hydroxyethyl cellulose. The process of interaction of ZnO NPs with polysaccharides was investigated. It was shown that the interaction of ZnO NPs with polysaccharides occurs through a charged hydroxyl group. In the experiment, a sample of a gel of ZnO NPs modified with hydroxyethyl cellulose was tested. It was shown that the gel of ZnO NPs modified with hydroxyethyl cellulose has a pronounced regenerative effect on burn wounds, which is significantly higher than that of the control group and the group treated with a gel of ZnO microparticles (MPs) and hydroxyethyl cellulose. It is also shown that the rate of healing of burn wounds in animals treated with gel of ZnO nanoparticles with hydroxyethyl cellulose (group 3) is 16.23% higher than in animals treated with gel of ZnO microparticles with hydroxyethyl cellulose (group 2), and 24.33% higher than in the control group treated with hydroxyethyl cellulose. The average rate of healing of burn wounds for the entire experimental period in experimental animals of group 3 is 1.26 and 1.54 times higher than in animals of group 2 and control group, respectively. An experimental study of a gel of ZnO NPs modified with hydroxyethyl cellulose has shown the effectiveness of its use in modeling the healing of skin wounds through primary tension.

## 1. Introduction

The history of the use of zinc as a biologically active mineral goes back to ancient times. Zinc ointment was used for skin diseases and to accelerate wound healing in ancient Egypt 5000 years ago. However, a serious study of the role of this mineral in biological processes began only in the middle of the twentieth century, after it was accidentally discovered that wounds began to heal much faster when a little zinc was added to the of diet rats that received burns [1].

Zinc is an important element for the functioning of a wide range of physiological functions of living organisms [2]. Zinc participates in carbohydrate metabolism through a zinc-containing hormone–insulin, and is also necessary for the absorption of vitamin A. Zinc also has anti-inflammatory properties [3,4,5]. Alkaline phosphatase dephosphorylates adenosine monophosphate to form adenosine, which, in turn, has pronounced anti-inflammatory properties and is important for interrupting the inflammatory phase of the wound process [6]. Sheferov et al. (2022) found that within 24 h after injury, the zinc content in the wound edges increases by 15–20%, reaching 30% during the period of maximum intensity of granulation tissue formation and epidermis proliferation [6]. Observed in the late stages of healing (10–21 days), the decrease in zinc content reflects a decrease in mitotic activity and maturation of scar tissue. Its average total content in the tissues of the human body is about 2–3 g. ZnO is listed by the FDA (USA) as a generally accepted safe material [7]. Zinc is a necessary element for our health and ZnO nanoparticles (NPs) also have a good biocompatibility with human cells [8].

Currently, an ongoing task for materials science is the development and research of gels based on ZnO. ZnO has a number of useful physical and chemical properties, which include high photosensitivity, the ability to effectively absorb UV radiation, antibacterial properties and protection from UV radiation. ZnO is widely used in modern science and technology. For example, the introduction of ZnO into cosmetic creams and gels increases their sunscreen and antibacterial properties [6]. The effectiveness of these products largely depends not only on the concentration of the active substance, ZnO, but also on the size of the particles themselves, their modification and the degree of polydispersity. It was also found that ZnO NPs can be used in drug delivery in the body [9] and for the treatment of dental diseases together with minocycline [10]. ZnO NPs exhibit biological activity against the bacteria Helicobacter suis, Helicobacter bizzozeronii, Helicobacter felis, and Helicobacter salomonis [11]. Babaevskaya et al. (2022) observed that there is a correlation between the polydispersity of ZnO particles and the effectiveness of the drug: the higher the particle dispersion, the more effective the drug based on these particles is [12]. Currently, metal NPs are being thoroughly studied and widely investigated as potential antimicrobial drugs. Antimicrobial activity of NPs, as is known, depends on the surface area that is in contact with microorganisms [13]. Antimicrobial wound dressings and various dressing materials are created on the basis of chitosan and heparin [14,15]. Due to their medicinal properties and a wide range of antimicrobial efficacy, ZnO NPs have made a breakthrough in the fight against infections during wound healing [16].

To date, many hydrogels with special functional inclusions from various biological substances have been studied. Essentially, polysaccharides demonstrate great potential for application in the field of nanomaterials due to the fact that they have high biocompatibility and non-toxicity [17,18,19,20,21,22]. Most biomaterials in nature exist in the form of polysaccharides. They can be distinguished not only by the nature of their constituent components, but also by the length of the molecular chains and the degree of branching of the chains. Xue and Luo (2021) created and evaluated a bandage made of a β-chitin-hydrogel/ZnO NPs composite as an alternative to existing dressings [23]. The bandages showed controlled swelling and antibacterial activity—in vivo evaluation on Sprague Dawley rats showed the fastest healing in the experiment. Hydrogel bandages containing ZnO NPs functionalized with heparin showed accelerated wound closure, repeated epithelization and skin regeneration [24]. Khan et al. (2022) functionalized arabinoxilane and graphene oxide (GO) using a hydrothermal method by crosslinking GO-arabinoxilane and polyvinyl alcohol (PVA) with tetraethylortosilicate (TEOS) to obtain multifunctional composite hydrogels [25]. In vivo studies were carried out using a full-layer mouse skin model. The authors observed an accelerated wound healing effect with improved vascularization and without any serious inflammation for seven days. Ghanadian et al. (2022) declared that the use of 10% topical gel of P. major leaf extract leads to accelerated healing of various peptic ulcer diseases [26]. It was found that the use of P. major gel leads to accelerated healing of diabetic foot ulcers and pressure sores, as P. major extract helps to reduce redness and wound size. Generally, the use of various biopolymers to accelerate wound healing is a very effective method at the present time, due to the good biocompatibility and antibacterial activity of new developing biofilms. Currently, hydrogel materials containing inclusions of chitosan, fibrin, graphene oxide, hypericin, azithromycin and other biopolymers are used [27,28,29,30,31,32,33,34,35].

The aim of this work was to synthesize ZnO NPs stabilized with various polysaccharides and to study their wound-healing properties.

## 2. Results and Discussion

### 2.1. Optimization of the Method of Synthesis of ZnO NPs Gels

The results of the effect of synthesis parameters on the dynamic viscosity of ZnO NPs gels are presented in Table 1.

As a result of the mathematical processing of experimental data, the dependences of the viscosity of ZnO gels on various synthesis parameters were obtained.

**Figure 1:**
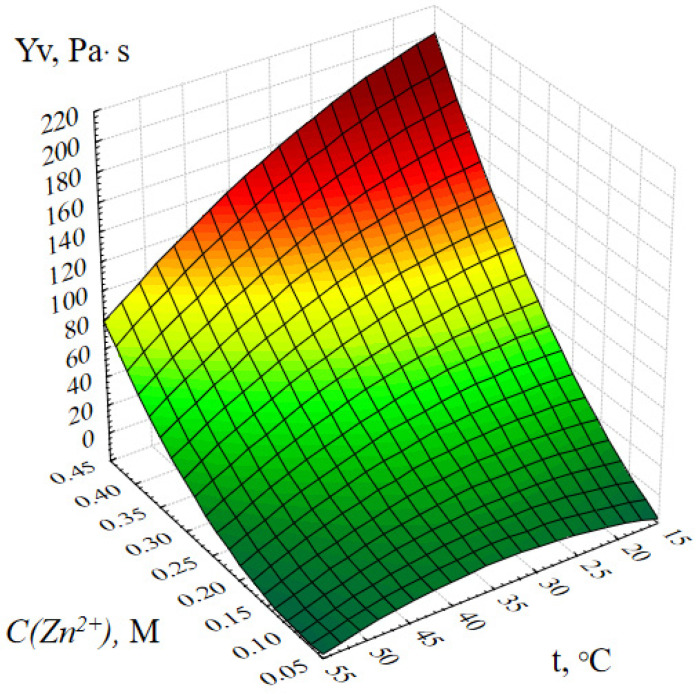
Response surface of the output parameter Y_v_ (dynamic viscosity of the ZnO gel) depending on the concentration of zinc acetate and the temperature of the reaction medium.

Figure 1 shows the response surface of the output parameter Y_v_ (dynamic viscosity of ZnO gels) depending on the concentration of zinc acetate and the temperature of the reaction medium under other optimal conditions.

The analysis of Figure 1 shows that the viscosity of ZnO gels increases almost linearly with an increase in the concentration of zinc acetate, a decrease in the temperature of the reaction medium and, conversely, falls almost to the value of the dynamic viscosity of pure solvent (water), with a decrease in C(Zn^2+^) and an increase in t. Moreover, during the experiment, it was observed that at temperatures above 40 ℃, the structure of ZnO gels was destroyed with the formation of a flake-like amorphous precipitate. This fact allows us to conclude that ZnO gels are thermally unstable (thermolabile), which correlates with results of Mocioiu et al. (2021) [23]. We assume this is due to the participation in the formation of the gel structure not only of ZnO NPs, but also of zinc compounds, such as its various hydroxoacetate complexes and hydroxides, which begin to break down at temperatures above 40 ℃, consequently leading to the destruction of the spatial structure of the entire gel.

**Figure 2:**
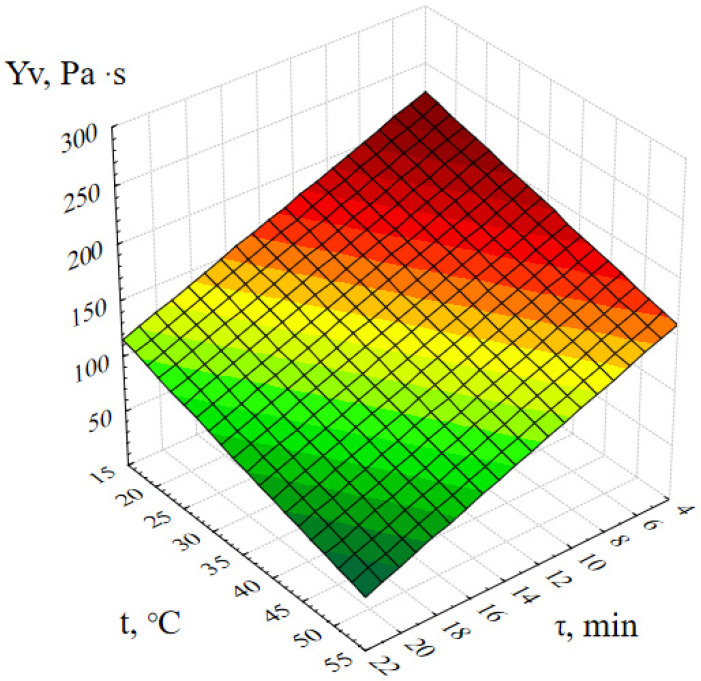
Response surface of the output parameter Y_v_ (dynamic viscosity of the ZnO gel) depending on the temperature of the reaction medium and the duration of synthesis.

The following graphical dependence (Figure 2) confirms that the most stable ZnO gels are formed at t = 20 ± 5 ℃, and the gels exposed to thermal effects during synthesis are completely destroyed after a while.

**Figure 3:**
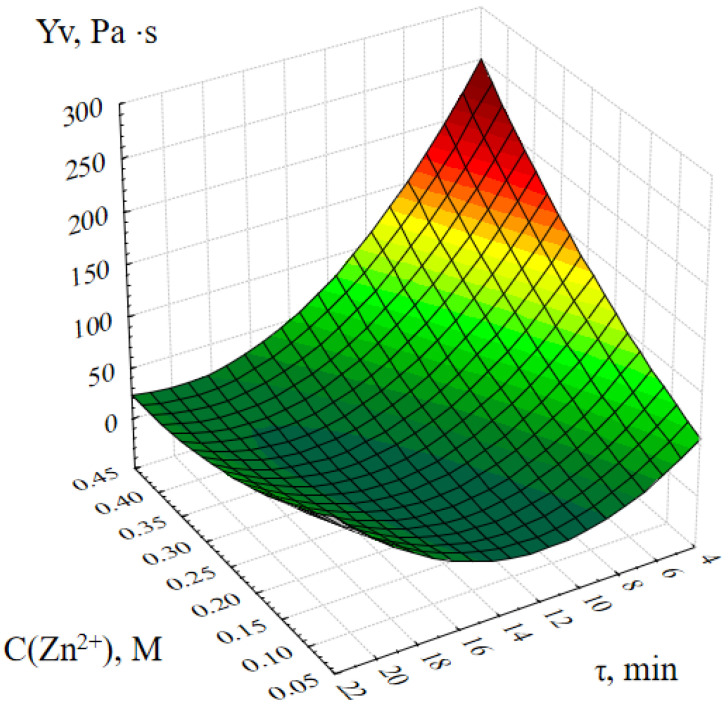
Response surface of the Y_v_ output parameter depending on the concentration of zinc acetate and the duration of synthesis.

Figure 3 shows the response surface of the output parameter Y_v_ (dynamic viscosity of the ZnO gel) depending on the concentration of zinc acetate in the reaction mass and the synthesis time.

The analysis of this graphical dependence shows that the dynamic viscosity of ZnO gels at the initial moment of time increases almost in direct proportion with an increase in the concentration of zinc ions C(Zn^2+^) in the initial reaction mixture. It is also important to note that the value of the dynamic viscosity of ZnO gels obtained at C(Zn^2+^) = 0.05 ÷ 0.2 M practically does not change with time, and when C(Zn^2+^) = 0.25 ÷ 0.45 M decreases sharply, it can be concluded that the spatial structure of such gels is not stable in time.

**Figure 4:**
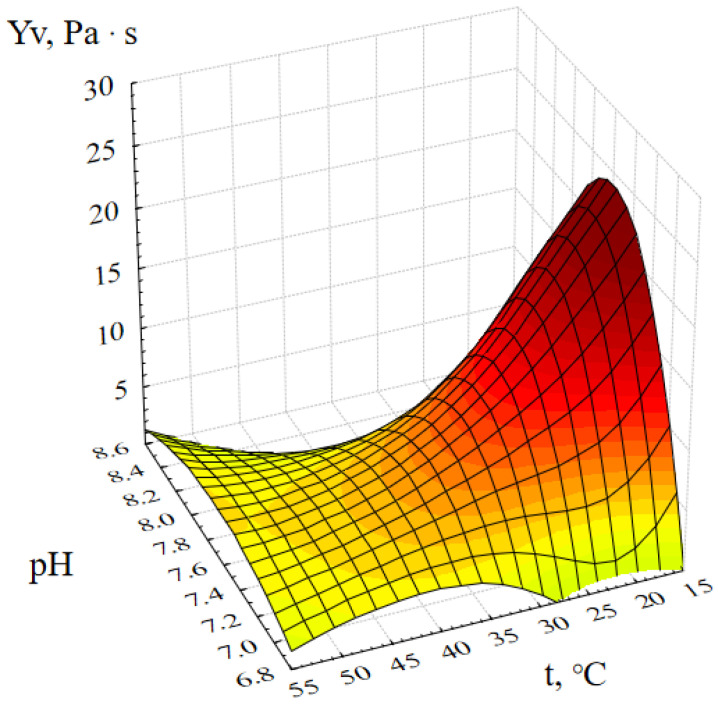
Response surface of the output parameter Y_v_ (dynamic viscosity of the ZnO gel) depending on the active acidity and temperature of the reaction medium.

Figure 4 shows the response surface of the output parameter Y_v_ (dynamic viscosity of the ZnO gel) depending on the active acidity and temperature of the reaction medium.

This graphical dependence confirms the previously presented response surfaces and shows the influence of another factor on the synthesis of ZnO gel–pH. Analysis of Figure 4 showed that the most viscous ZnO gels are formed at room temperature and at pH ≈ 8. This is explained by the fact that complete deposition of zinc ions occurs only at pH = 8 [24]. Thus, at pH < 8, Zn^2+^ ions are still present in the reaction mass, and at pH > 8 oxide, hydroxide and various zinc acetate complexes begin to dissolve with the formation of zinc anions [Zn(OH)_4_]^2−^ [25,26].

Based on the analysis of the data obtained, it can be concluded that the most stable complex ZnO gels are formed at room temperature, pH = 8 and molar concentration of zinc C(Zn^2+^) = 0.05 ÷ 0.2 M.

The essence of the processes is as follows: when an ammonia solution is added to an aqueous solution of zinc acetate to pH = 7, a gel of zinc hydroxide Zn(OH)_2_ is formed, which, when the pH rises to ~ 8, turns into ZnO. With further addition of an ammonia solution and an increase in the acidity of the reaction medium, zinc hydroxide and oxide begin to dissolve to form amino- (Zn(NH_3_)_2_^2+^) and zinc hydroxocomplexes ([Zn(OH)_4_]^2−^) [27,28]. As a result, it can be concluded that ZnO gels synthesized by precipitation with ammonia from soluble zinc salts contain in their composition, in addition to the formed phase of ZnO and hydroxide, various zinc complex compounds (amino and aqua complexes).

**Figure 5:**
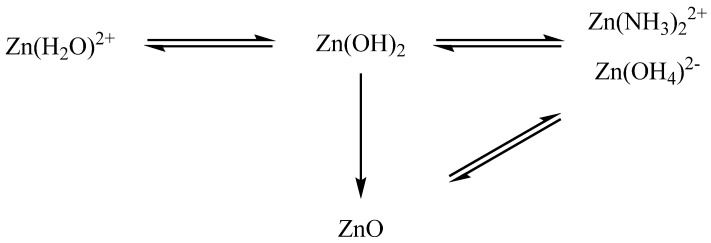
Scheme of chemical transformations occurring during the synthesis of complex ZnO gels.

The chemical processes that occur during the synthesis of ZnO gels are schematically presented in Figure 5.

### 2.2. Investigation of Gels of ZnO NPs Modified with Polysaccharides

**Figure 6:**
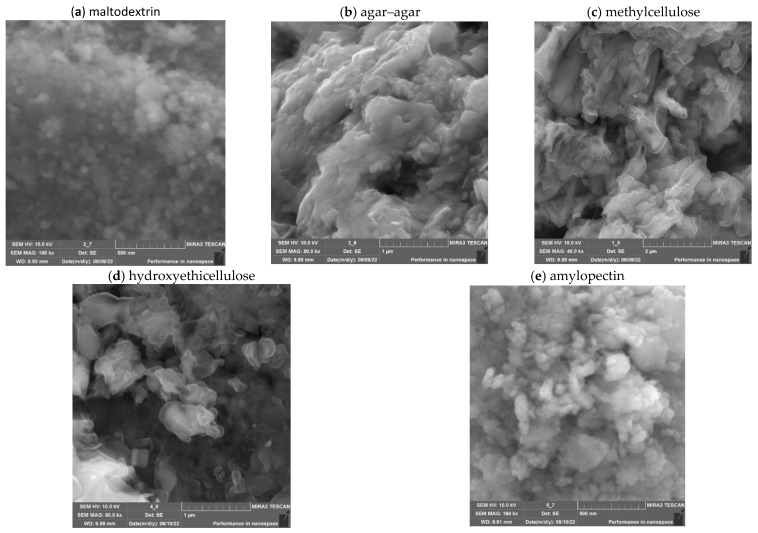
SEM micrographs of nanoscale ZnO stabilized with polysaccharides.

At the next stage, the effect of the addition of polysaccharides (maltodextrin, agar–agar, methylcellulose, hydroxyethicellulose, amylopectin) on the structure and rheological properties of nanoscale ZnO gels was investigated. The microstructure of nanoscale ZnO gels was studied using scanning electron microscopy (SEM). The resulting SEM micrographs are shown in Figure 6.

Analysis of SEM micrographs of nanoscale ZnO showed that the samples of ZnO NPs gels consist of irregularly shaped aggregates. In the agar–agar stabilized sample, the aggregates have a lamellar shape. In turn, the surface of ZnO NPs stabilized with methylcellulose and hydroxyethylcellulose is represented by irregularly shaped particles that are assembled into aggregates with sizes ranging from 150 to 1400 nm. In addition, the microstructure of samples with maltodextrin and amylopectin is represented by spherical particles, the diameter of which ranged from 30 to 70 nm.

**Figure 7:**
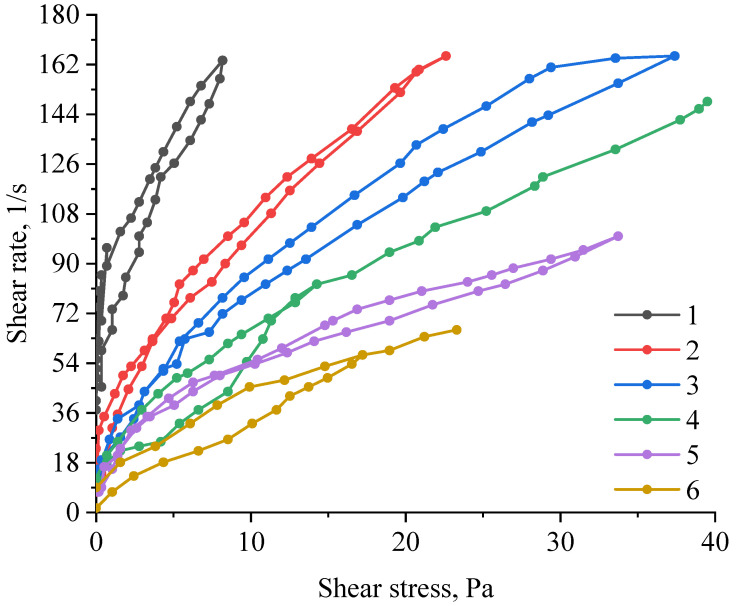
Dependence of the shear rate on the shear stress of samples of gels of ZnO with polysaccharides: 1—ZnO with amylopectin; 2—ZnO with agar–agar; 3—ZnO with hydroxyethyl cellulose; 4—ZnO with maltodextrin; 5—ZnO with cellulose; 6—ZnO gel without polysaccharide.

At the next stage, the rheological properties of ZnO NPs gels were investigated. The obtained dependences of the shear rate on the shear stress are shown in Figure 7.

The analysis of the obtained dependences showed that the addition of polysaccharides to the samples of ZnO gels significantly affects their rheological properties. It was also found that the nature of the polysaccharide significantly affects the type of dependence of the shear rate on the shear stress. A slight hysteresis loop is observed in a sample of ZnO gel without polysaccharides. A similar dependence is observed in all samples of ZnO NPs gels except for the sample containing hydroxyethyl cellulose. A significant hysteresis region is present in the sample modified with hydroxyethyl cellulose, which is explained by the preservation of residual deformation after a strong weakening of the structure under the influence of previously applied stress [29]. The presence of ascending and descending curves of the hysteresis loop indicates that the sample under study has rheopexic properties.

**Figure 8:**
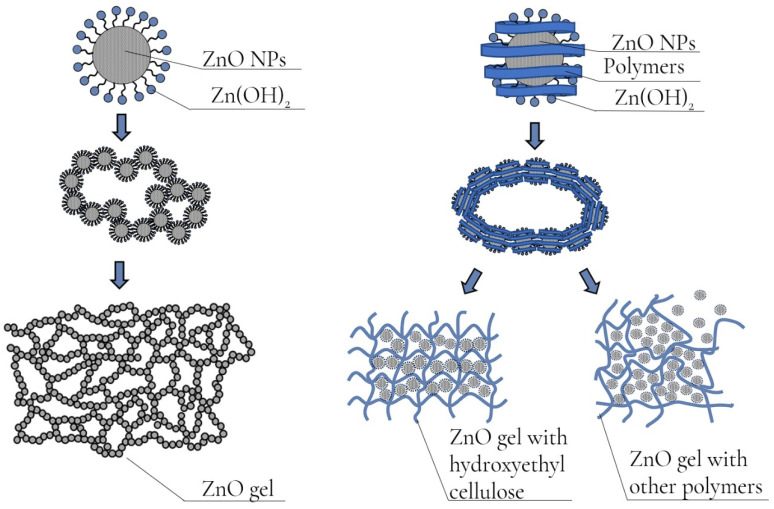
Diagram of the structure of gels of ZnO NPs with polysaccharides.

Analysis of the data obtained showed that the optimal polysaccharide for the synthesis of ZnO NPs gels is hydroxyethyl cellulose, which forms a gel with an ordered structure. In the case of using other polysaccharides, the formation of a disordered gel structure is observed, which is confirmed by the data of the rheological properties study (Figure 8).

At the next stage, the process of polysaccharide interaction with the surface of nanoscale ZnO was investigated using computer quantum chemical modeling in the QChem program using the IQmol molecular editor. The data of quantum chemical calculations of molecular complexes “ZnO-polysaccharide” are presented in Table 2.

As a result of the simulation, the value of the absolute chemical hardness ɳ is calculated. This value characterizes the stability of the system, preventing its change.

**Figure 9:**
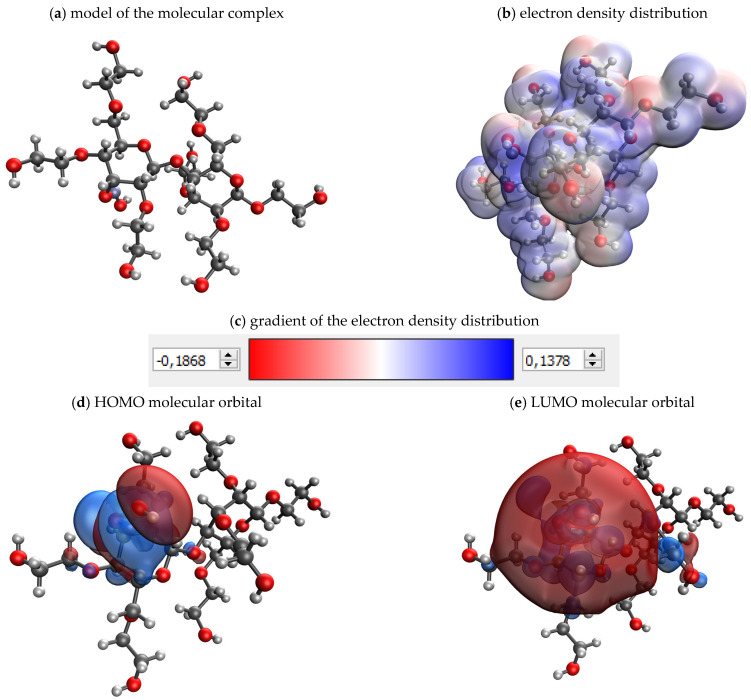
Results of quantum chemical modeling of the molecular system “ZnO-hydroxyethyl cellulose”.

The analysis of the obtained data showed that the most stable system is the molecular system “ZnO-hydroxyethyl cellulose”, for which the value of absolute chemical hardness (ɳ) was 0.193 eV (Figure 9).

**Figure 10:**
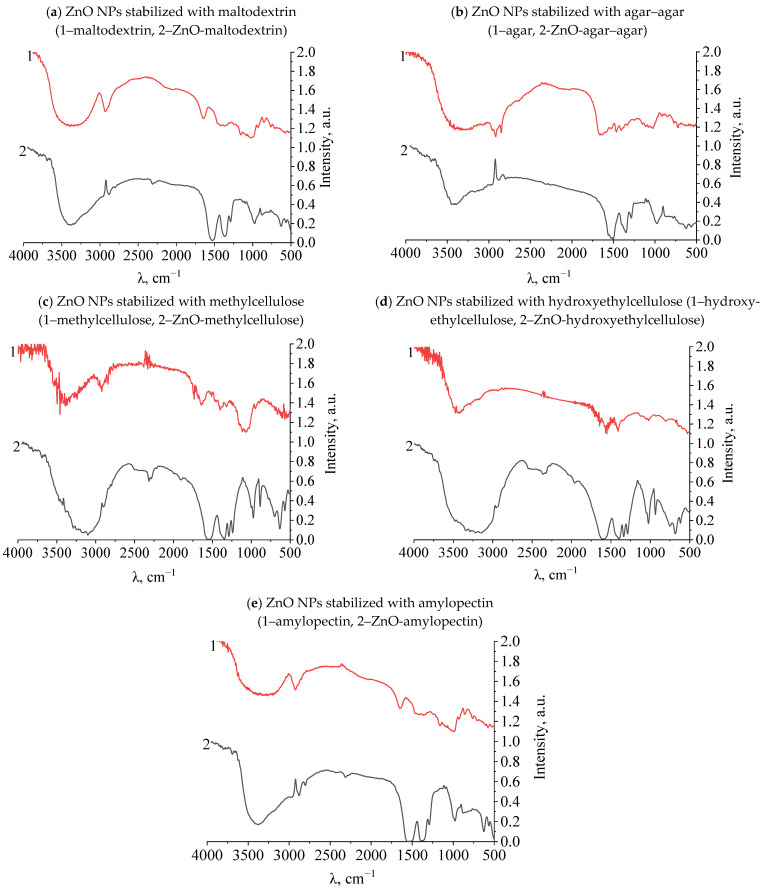
IR spectra of stabilized ZnO NPs.

At the next stage, the presence of fluctuations in the bonds of functional groups was studied in samples of gels of ZnO NPs modified with polysaccharides. The study was carried out using the infrared spectroscopy method on the FSM-1201 device. The results are presented in Figure 10a–d and in Table 3.

As a result of the analysis of IR spectra, it was found that in the spectra of all polysaccharides in the range from 1350 to 1450 cm^−1^, there is a significant drop in the intensity of the bands characterizing the deformation vibrations of the ionized charged group–OH. Thus, it was established that the interaction of ZnO NPs occurs through the charged hydroxyl group of the polysaccharide [30,31,32]. The results of IR spectroscopy are consistent with the results of computer quantum chemical modeling.

### 2.3. Approbation of a Gel of ZnO NPs Modified with Hydroxyethyl Cellulose

As a result of modeling a burn wound, burns of the III B degree were formed, which had a rounded shape; the bottom of the wounds was bright red, with a brown tinge in places. The edges of the wound were slightly overhanging pieces of red–brown soft tissue. There was a hyperemia zone 0.5–0.8 cm wide around the wound.

On the third day, in animals of all experimental groups, a scab began to be rejected at the edges of the wound surface with varying intensity, and weak epithelization of the wound edges was observed. The demarcation lines around the wounds in Group 3 (gel of ZnO NPs modified with hydroxyethyl cellulose) were in the range of 2–3 mm. The weakest regeneration processes were observed in Group 2 (gel of ZnO MPs and hydroxyethyl cellulose). The demarcation line in Group 2 reached 8 mm. There was a reduction in the area of burn wounds and gradual rejection of the scab, marking marginal epithelization of the burn wound.

In Group 1 (hydroxyethylcellulose gel) and Group 3, deep wounds were formed in all animals in place of scabby crusts, along the edges of which epithelization was slowly going on, and an increasing hyperemia zone was noted around the wound. In Group 2, where the animals were applied with zinc ointment, extensive edema zones were noted around the wounds, reaching a diameter of 25 mm, irregular shape and fuzzy edges. By the 6th day, the burn scab rejection occurred in the animals of Group 3. The scab in the experimental groups was dry, thin. Regeneration processes were actively going on under it. There was an increased marginal epithelization without the development of concentric contraction of wounds.

**Figure 11:**
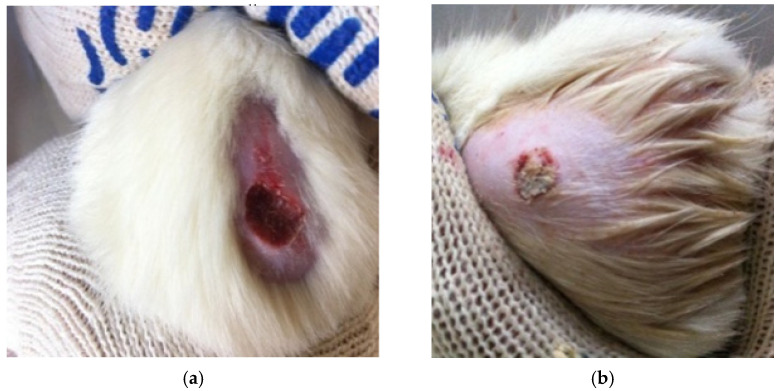
Photos of burn wounds of laboratory animals of group 1: (**a**)—3rd day of the experiment, (**b**)—10th day of the experiment.

On the 10th day of the experiment, a scab was removed from the burn surfaces (two individuals from each group) (Figure 11). A clean and pink bottom, located at the level of the edges of healthy skin, was observed only in animals of Group 3, and positive reparative processes were noted, including the restoration of microcirculation in burn wounds. In other groups, the bottom was deep, uneven, and the scab was removed with difficulty; the repair processes under it were slow with the risk of developing concentric contraction and scarring. It should be noted that at the beginning of the experimental period, the experimental animals of Group 2 had a distinct allergic reaction to a gel of ZnO MPs and hydroxyethyl cellulose, which “slowed down” the healing process of the skin, and by the end of the experimental period, when treating burn wounds with zinc ointment, complete regeneration did not occur.

**Figure 12:**
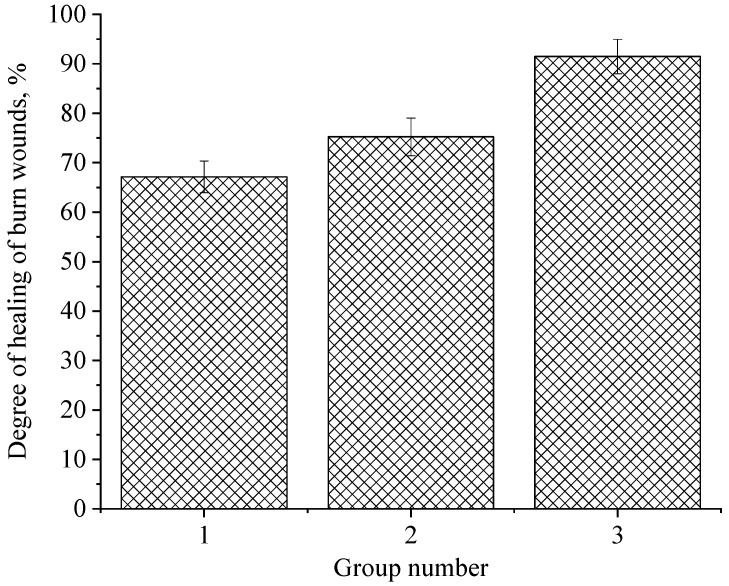
The degree of healing of burn wounds in experimental animals: 1—hydroxyethylcellulose gel; 2—gel of ZnO MPs and hydroxyethylcellulose; 3—gel of ZnO NPs modified with hydroxyethylcellulose (*p* = 0.05).

At the next stage, the degree of healing of burn wounds on the 21st day of the experiment was evaluated. The data obtained are presented in Figure 12.

Analysis of Figure 12 allows us to conclude that the degree of healing of burn wounds in experimental animals of Group 3 (gel of ZnO NPs modified with hydroxyethyl cellulose) is 16.23% higher than in laboratory animals of Group 2 (gel of ZnO MPs and hydroxyethyl cellulose) and 24.33% higher than in Group 1. This fact indicates the effectiveness of the developed gel of ZnO NPs modified with hydroxyethyl cellulose.

**Figure 13:**
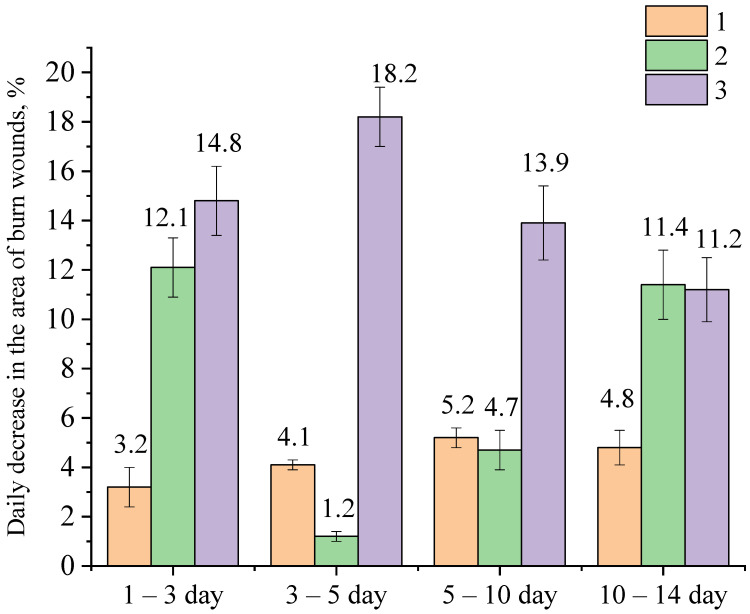
The rate of healing of burn wounds in experimental animals: 1—hydroxyethylcellulose gel; 2—gel of ZnO MPs and hydroxyethylcellulose; 3—gel of ZnO NPs modified with hydroxyethylcellulose.

The results of calculations of the healing rate of burn wounds in experimental animals are shown in Figure 13.

Analysis of the obtained data presented in Figure 13 showed that the rate of healing of burn wounds in experimental animals of Group 3, to which the gel of ZnO NPs modified with hydroxyethyl cellulose was applied to the wound surface, was higher than in Group 1 and Group 2 almost up to the 10th day of the experiment. On the 1st–3rd day of the experiment in animals of Group 3, the value of the healing rate of burn wounds was 0.43 cm^2^/day, which is 1.34 and 6.14 times higher than in animals of Group 2 and 1, respectively. On the 3rd–5th day, in animals of Group 3, the value of the healing rate of burn wounds was 0.31 cm^2^/day, which is 6.2 and 7.75 times higher than in animals of Group 2 and Group 1, respectively. On the 5th–10th day, in experimental animals, to whose wound surfaces gel of ZnO NPs modified with hydroxyethyl cellulose was applied, the value of the healing rate of burn wounds was 0.18 cm^2^/day, which is 1.8 and 1.29 times higher than in animals of Group 2 and Group 1, respectively. From all of the above, it follows that the rate of healing of burn wounds in experimental animals of Group 3 gradually decreases by the end of the experiment. The decrease in the average rate of healing of experimental wounds in Group 3 by the 10th–14th days is due to the fact that 60–70% of animals in this group had almost complete healing of burn wounds by this time.

**Figure 14:**
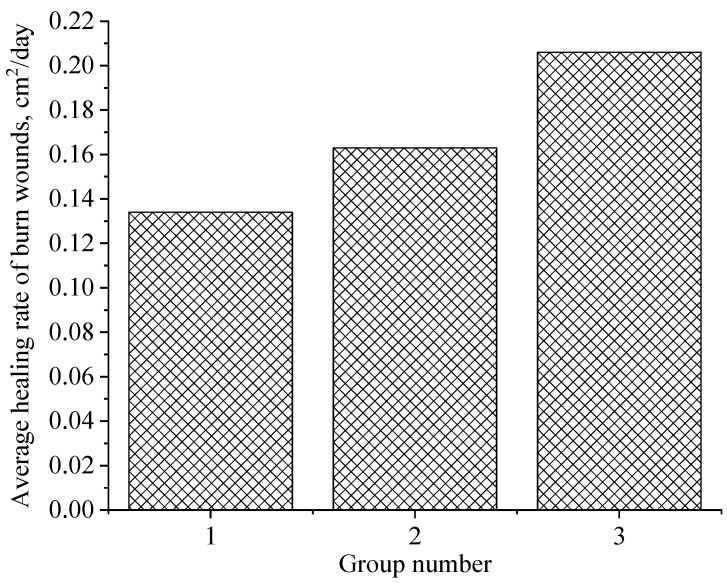
The average rate of healing of burn wounds over the entire experimental period in animals: 1—hydroxyethylcellulose gel; 2—gel of ZnO MPs and hydroxyethylcellulose; 3—gel of ZnO NPs modified with hydroxyethylcellulose.

The following Figure 14 shows the values of the average rate of healing of burn wounds for the entire experimental period in animals of the studied groups.

The analysis of Figure 14 allows us to conclude that the average rate of healing of burn wounds for the entire experimental period in experimental animals of Group 3 is 1.26 and 1.54 times higher than in animals of Group 2 and Group 1, respectively.

Thus, the analysis of changes in the area of wounds in dynamics in experimental animals showed that the gel of ZnO NPs modified with hydroxyethyl cellulose has a pronounced regenerative effect of burn wounds, which is significantly higher than in the Group 1 and Group 2. The use of ZnO NPs with a large specific surface area in the composition of the gel makes it possible to enrich the cells and tissues of the damaged wound surface with zinc ions in the right amount. Due to the normalization of the level of zinc intake into the cells of the damaged wound surface, the normalization of thymidine kinase, the rate of the limiting enzyme of DNA synthesis and cell replication, also occurs, which was confirmed and described in other works [33,34,35].

At the next stage, histological studies were performed. In animals of the experimental group, during the same period, a large fragmented scab covered the wound partially, and was sometimes separated from the underlying tissues throughout. Mature granulation tissue containing various hematogenic and tissue cellular elements was located in the center of the wound, and single macrophages, fibroblasts and collagen fibers appeared from the edges of the wound. The growth of the newly formed epithelium was observed, and the length of the regenerate was 574.5 ± 12.46 µm in the experimental group versus 589.01 ± 14.58 µm in the control group. Hypertrophy was detected at the border with the intact epidermis in both groups.

**Figure 15:**
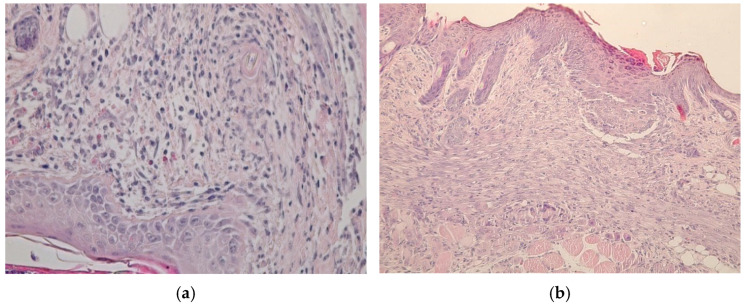
Results of histological studies on the 5th day of the experiment: (**a**) Control group. Stained with hematoxylin and eosin. Magnification ×400; (**b**) Experimental group. Stained with hematoxylin and eosin. Magnification ×200.

Five days after the beginning of healing, the remains of a fragmented scab remained in the center of the injury in the animals of the control group. A small area of granulation tissue containing round-cell elements was localized under the scab. At the border with the intact skin, the epithelium was sharply hypertrophied, and the regenerate was distinguished by the formation of small outgrowths of the basement membrane into the underlying tissue. Formed granulation tissue with leukocyte infiltrate in the area of wound defect of the dermis occurred in the control group (Figure 15a). In animals of the experimental group, partial or complete epithelization of the lesions occurred at the same time. In some rats, an even epithelial layer formed on the wound surface, consisting of 8–10 rows of cells with an even basement membrane that does not form outgrowths into the thickness of the dermis, shown in Figure 15b. Its length was 175 ± 9 versus 236 ± 8 µm in the control group (*p* < 0.05). Under the epithelium, the wound defect was filled with mature granulation tissue with pronounced vascularization and a characteristic horizontal arrangement of fibroblasts around the vessels.

**Figure 16:**
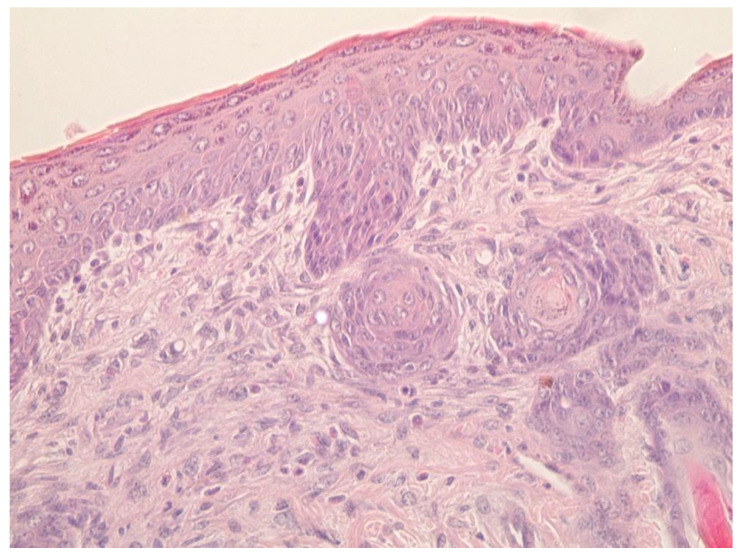
The structure of the newly formed epidermis in the control group (stained with hematoxylin and eosin. Magnification ×400).

After seven days, the differences we noted earlier presented especially clearly. In the animals of the control group, complete epithelization of experimental lesions was observed. The newly formed epithelial layer, consisting of several rows of cells, covered the entire area of the defect for 634 ± 22. Its basement membrane had an uneven configuration, but no outgrowths were observed in the underlying tissue and the formation of hair follicles and sebaceous glands did not occur. Under the epithelium there was connective tissue with typical cellular structures, shown in Figure 16.

**Figure 17:**
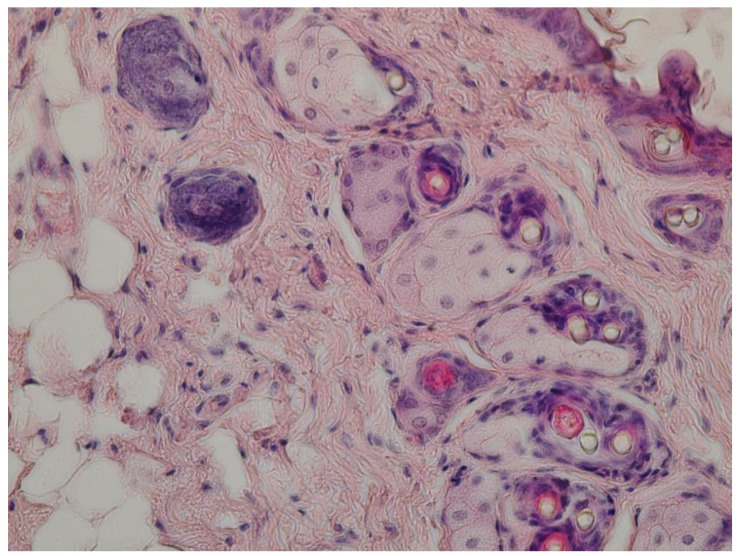
Multi-stem hair follicles in experimental group (stained with hematoxylin and eosin. Magnification ×400).

In animals of the experimental group, the wound process ended with the formation of an organ-specific regenerant. This was evidenced by the complete epithelialization of wounds, pronounced contraction of the lesion area, expressed in a significantly shorter length of the regenerate (641 ± 25 in experimental group vs. 664 ± 28 µm in control group) and neoplasm of hair follicles and sebaceous glands (Figure 17).

Thus, an experimental study of gel of ZnO NPs modified with hydroxyethyl cellulose has shown the effectiveness of its use in modeling the healing of skin wounds through primary tension. The inclusion of ZnO NPs in the composition leads to a less pronounced and rapidly relieving process of inflammation, which is a morphological substrate for the formation of granulation tissue in the area of injury. Active angiogenesis and proliferation of fibroblasts accompanying the formation of granulations, their subsequent transformation into connective tissue, and an increase in the rate of epithelialization of the injury zone are essentially a reflection of the transition of the wound healing process from the phase of inflammation to the phase of proliferation.

## 3. Conclusions

For the experiment, a method was developed for the synthesis of ZnO NPs gels. It was found that the most stable gels of ZnO NPs are formed at room temperature, pH = 8 and a molar concentration of zinc C(Zn^2+^) = 0.05–0.2 M. It was shown that the addition of polysaccharide significantly affects the rheological properties and microstructure of ZnO NPs gels. It was shown that the optimal polysaccharide for the synthesis of ZnO NPs gels is hydroxyethyl cellulose. It was shown that the microstructure of gel of ZnO NPs stabilized with hydroxyethyl cellulose is represented by irregularly shaped particles that are assembled into aggregates with sizes from 150 to 1400 nm. The process of interaction of ZnO NPs with polysaccharides was investigated. It was determined that the interaction of ZnO NPs with polysaccharides occurs through a charged hydroxyl group.

During the experiment, we tested a sample of the gel of ZnO NPs modified with hydroxyethyl cellulose. It was shown that the gel of ZnO NPs modified with hydroxyethyl cellulose has a pronounced regenerative effect of burn wounds, which is significantly higher than that of the control group and the group treated with a gel of ZnO MPs and hydroxyethyl cellulose. It was also shown that the rate of healing of burn wounds in animals treated with gel of ZnO nanoparticles with hydroxyethyl cellulose (group 3) is 16.23% higher than in animals treated with gel of ZnO microparticles with hydroxyethyl cellulose (group 2), and 24.33% higher than in the control group treated with hydroxyethyl cellulose. The average rate of healing of burn wounds for the entire experimental period in experimental animals of group 3 is 1.26 and 1.54 times higher than in animals of group 2 and control group, respectively. An experimental study of a gel of ZnO NPs modified with hydroxyethyl cellulose has shown the effectiveness of its use in modeling the healing of skin wounds through primary tension.

## 4. Materials and Methods

### 4.1. Substances and Reagents

Zinc acetate II (Lenreactive, St. Petersburg, Russia), 25% ammonia solution (HIMEKS, Pyatigorsk, Russia), maltodextrin (Lenreactive, St. Petersburg, Russia), agar–agar (Lenreactive, St. Petersburg, Russia), methylcellulose (TD Himmed, Moscow, Russia), hydroxyethicellulose of the B30K brand (HimSpektr, Krasnodar, Russia), amylopectin (Lenreactive, St. Petersburg, Russia), and 0.9% sodium chloride solution (GROTEX, St. Petersburg, Russia) were obtained.

### 4.2. Synthesis of ZnO NPs Gels

Synthesis of ZnO NPs gels was carried out using the sol–gel method. At the first stage, 8.76 g of zinc acetate 2-aqueous (Zn(CH_3_COO)_2_·2H_2_O) was weighed on analytical scales. Then, the suspension was quantitatively transferred to a beaker and 200 mL of distilled water was added. A 12.5% ammonia solution was added to the resulting solution to obtain pH = 8. The resulting mixture was left to stir for 30 min until the gel was formed. The resulting ZnO NPs gel was centrifuged at 2500 rpm for 5 min and washed 3 times with distilled water. The resulting ZnO precipitate was dried at a temperature of 80 ℃.

### 4.3. Synthesis of Gels of ZnO NPs Modified with Polysaccharides

**Figure 18:**
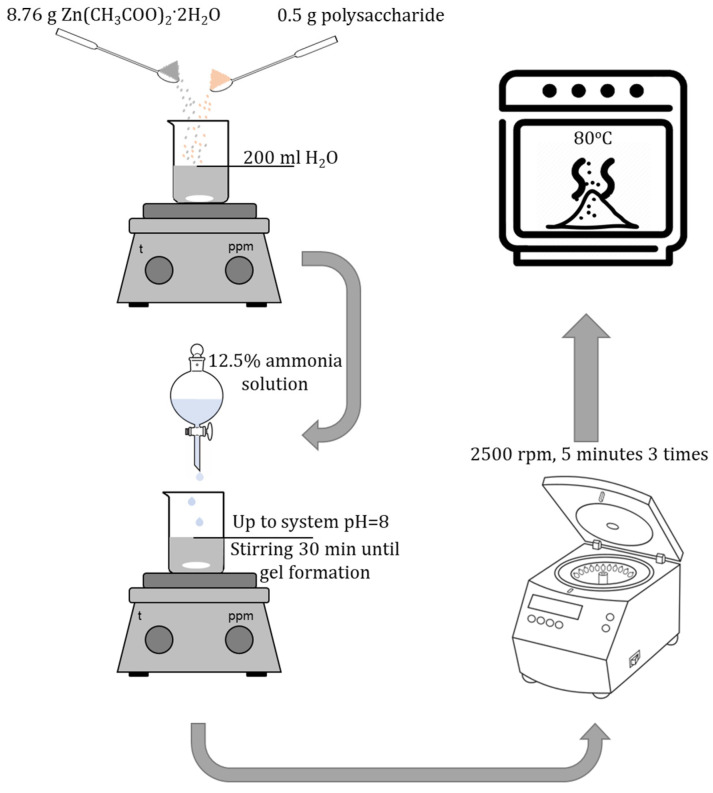
Scheme of synthesis of gels of ZnO NPs modified with polysaccharides.

Synthesis of gels of ZnO NPs modified with polysaccharides was carried out using the sol–gel method. At the first stage, 8.76 g of zinc acetate 2-aqueous (Zn(CH_3_COO)_2_·2H_2_O) and 0.5 g of polysaccharide were weighed on analytical scales. Maltodextrin, agar–agar, methylcellulose, hydroxyethylcellulose, amylopectin were used as polysaccharides. The suspension was then quantitatively transferred to a beaker and 200 mL of distilled water was added. A 12.5% ammonia solution was added to the resulting solution to obtain pH = 8. The resulting mixture was left to stir for 30 min until the gel was formed. The resulting ZnO NPs gel was centrifuged at 2500 rpm for 5 min and washed 3 times with distilled water. The resulting ZnO precipitate was dried at a temperature of 80 ℃ (Figure 18).

### 4.4. Characteristics of ZnO NPs Gels

To study the obtained samples, an IR spectrometer FSM 1201 (Infraspec, Saints Petersburg, Russia) with Fourier-transform was used. Research parameters:

- measurement range: from 400 to 4000 cm^−1^.
- measurement step: 1 cm^−1^.

Samples of ZnO NPs were examined using scanning electron microscopy on a MIRA-LMH scanning electron microscope with the AZtecEnergy Standard/X-max 20 (standard) elemental composition determination system (Tescan, Brno-Kohoutovice, Czech Republic).

Quantum chemical modeling was carried out in the Q-Chem software using the IQmol molecular editor (Q-Chem Inc., Pleasanton, CA, USA). Simulation parameters: Calculation–Energy, method–M06, Basis–6-31G*, Convergence–4, Force field–Chemical. To simplify calculations, free bonds were hydrogenated.

The study of rheological properties and measurement of the dynamic viscosity of ZnO NPs gels was carried out on the IKA ROTAVISC me-vi advanced rotary viscometer (IKA-Werke GmbH & Co. KG, Staufen, Germany).

### 4.5. Investigation of the Effect of Synthesis Parameters on the Dynamic Viscosity of ZnO NPs Gels

Preliminary experiments and analysis of the literature data revealed the factors (variable parameters) that have the greatest impact on the synthesis of ZnO gels [36,37,38,39]. The following parameters are selected as variables:

1. Molar concentration of zinc acetate, M;
2. Active acidity of the reaction medium (pH);
3. Duration of synthesis, minutes;
4. Temperature of the reaction medium, ℃.

The variable parameters during the experiments took the values indicated in Table 4.

An experiment-planning matrix was created for the synthesis of ZnO gels with different parameters. The output parameter was the dynamic viscosity of ZnO gels–Y_v_, MPa·s. The planning matrix was obtained with the method of Greek-Latin squares and is presented in Table 5.

**Figure 19:**
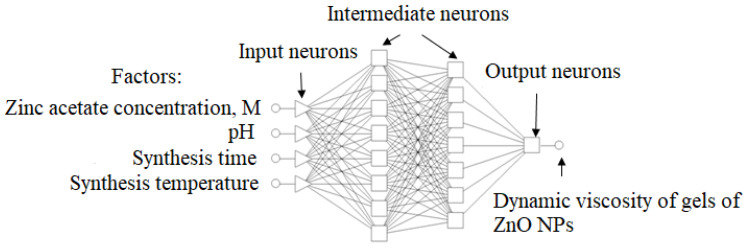
Multilayer perceptron.

The experimental data were processed using the Statistica 10.0 software package (StatSoft, Tulsa, OK, USA). The obtained results were processed using the Statistica Neural Networks v.4.0e application software package. The multilayer perceptron is shown in Figure 19.

### 4.6. Investigation of Wound-Healing Ability of ZnO NPs Gels

An experiment to study the effect of an ointment composition based on ZnO NPs gels on the healing process of burn wounds was carried out on mongrel white rats aged 6 months and weighing up to 250 g. To test the ointment composition, clinically healthy laboratory animals were used, which were kept in the same vivarium conditions on a standard food regime. For the research, three groups with ten rats in each were formed:

Group 1–hydroxyethyl cellulose gel;

Group 2–gel containing ZnO MPs and hydroxyethyl cellulose;

Group 3–gel of ZnO NPs modified with hydroxyethyl cellulose;

The duration of the experiment was 21 days, during 7 of which the animals were kept in quarantine. A day before the simulation of thermal burn on the side surface of the rat’s body, 4 × 4 cm skin areas were shaved with a safety razor. Having previously injected 5–10 mL of 0.9% NaCl solution under the skin for the convenience of shaving, the skin was stretched and no damage occurred during shaving.

Thermal burn modeling was carried out in accordance with generally accepted methods [37,38,39,40]. When burns were applied, an empty glass tube with an inner diameter of 22 mm (cross-sectional area of 4 cm^2^) and a length of 15 cm was filled with hot water, placed vertically in boiling water at 2/3 of the height, heated for 1 min, filled over the edge at 2/3 of the height and, in a vertical position, brought into close contact with the exposed skin of the animal for 10 s. Its area is about 8–9% of the entire body surface. To calculate its values in a rat, the Meeh Formula (1) was used [40]:

(1) $$S_{b}=k\times \frac{2}{3}W\;,$$

where:

*S_b_* is the surface of the body, cm^2^;

*W* is the body weight of the animal, kg;

*k* is the Meeh constant (9.46).

Twice a day, the experimental animals were examined, the wound surface was treated and the healing indicators were recorded. During the entire experimental period, we monitored the burn surface on the 1st, 3rd, 5th, 10th and 14th days. The tested substances were used at a dose of 10 mg/cm^2^. A saline solution was used as a control substance. On the next day after the simulation of the burn, the objects were monitored.

For the purpose of a more detailed analysis of the dynamics of regeneration of burn wounds in experimental animals of all experimental groups, the following parameters were calculated:

The rate of wound healing was determined using the Markaryan and Sarkisyan Formula (2) [41]:

(2) $$U=\frac{S-S_{n}}{n},$$

where:

*U* is the healing rate of the wound area per day, cm^2^/day;

*S* is the area of the wound at the previous measurement, cm^2^;

*S_n_* is the area of the wound at this measurement, cm^2^;

*n* is the number of days, day.

The average rate of healing of burn wounds for the entire experimental period was determined according to the Formula (3):

(3) $$U_{\mathrm{av}}=\frac{S_{1}-S_{2}}{\tau },$$

where:

U_av_ is the average healing rate of the wound area for the entire experimental period, cm^2^/day;

S_1_ is the area of the wound at the initial moment of the experiment, cm^2^;

S_2_ is the area of the wound at the final moment of the experiment, cm^2^;

τ is the duration of the experiment, day.

### 4.7. Histological Research

Histological sections with a thickness of 5–6 μm were carried out on a sledge microtome MS-2 (ATMpractica, Saints Petersburg, Russia). Finished sections were stained with hematoxylin and eosin, followed by histopathological analysis. Histological micropreparation was evaluated using a laboratory microscope of the Axio Imager 2 (A2) research class (Carl Zeiss Microscopy, Oberkochen, Germany) at magnifications of ×200 and ×400 with image fixation using a specialized AxioCam MRc5 camera (Carl Zeiss Microscopy) and Zen V.2 software (Carl Zeiss Microscopy) according to protocol from our previous work [42].

The experiment was carried out for two groups:

(1) A group treated with a gel of ZnO MPs and hydroxyethyl cellulose (control group);
(2) A group treated with a gel of ZnO NPs modified with hydroxyethyl cellulose (experimental group).

## Figures and Tables

**Table 1:** Numerical values of variable parameters and response function values.

No.	a	b	c	d	Viscosity, MPa·s
1	0.1	7.0	5	20	1.658
2	0.1	7.5	10	30	2.019
3	0.1	8.0	15	40	1.931
4	0.1	8.5	20	50	2.956
5	0.2	7.0	10	40	2.809
6	0.2	7.5	5	50	8.061
7	0.2	8.0	20	20	4.422
8	0.2	8.5	15	30	2.081
9	0.3	7.0	15	50	7.255
10	0.3	7.5	20	40	10.535
11	0.3	8.0	5	30	66.00
12	0.3	8.5	10	20	4.606
13	0.4	7.0	20	30	13.20
14	0.4	7.5	15	20	6.633
15	0.4	8	10	50	44.333
16	0.4	8.5	5	40	34.238

**Table 2:** Results of quantum chemical modeling.

Model	Energy, kcal/mol	HOMO, eV	LUMO, eV	ɳ, eV
ZnO-maltodextrin	−3140.336	−0.262	0.097	0.1795
ZnO-agar–agar	−2989.534	−0.285	0.085	0.185
ZnO-methylcellulose	−3260.501	−0.263	0.106	0.1845
ZnO-hydroxyethyl cellulose	−4210.149	−0.297	0.089	0.193
ZnO-amylopectin	−4354.377	−0.249	0.119	0.184

Energy—the total energy of the system, HOMO—the highest occupied molecular orbital, LUMO—the lowest unoccupied molecular orbital, ɳ—the chemical rigidity of the system.

**Table 3:** IR spectroscopy results.

Λ,cm^−1^	Bonds Fluctuations	λ,cm^−1^	Bonds Fluctuations	λ,cm^−1^	Bonds Fluctuations	λ,cm^−1^	Bonds Fluctuations
Maltodextrin		ZnO-maltodextrin		Agar–agar		ZnO-agar–agar	
3335	C–H	3399	C–H	3275	C–H	3414	C–H
2912	C–H	2880	C–H	3080	C–H	2893	OH^−^
1627	C–C	2899	C–H	2956	C–H	1564	CH_3_
1437	OH^−^	1529	C–C	2916	C–H	1496	OH^−^
1388	CH_3_	1401	OH^−^	1656	C–C	1342	CH_3_
1211	CH_3_	1296	CH_3_	1462	OH^−^	1287	CH_3_
1140	CH_2_	978	CH_3_	1415	OH^−^	1134	CH_2_
1058	CH_2_	883	CH_3_	1332	OH^−^	1098	CH_2_
989	CH_3_	637	C–H	1263	CH_3_	980	CH_3_
918	CH_3_	567	Zn–O	1159	C–O	875	CH_3_
835	CH_3_	-	-	1122	C–O	782	C–H
752	C–H	-	-	1084	CH_2_	631	C–H
683	C–H	-	-	1030	CH_2_	565	Zn–O
-	-	-	-	767	CH_2_, CH_3_	-	-
-	-	-	-	729	CH_2_, CH_3_	-	-
**Methylcellulose**		**ZnO-methylcellulose**		**Hydroxyethyl-cellulose**		**ZnO-hydroxyethylcellulose**	
3478	C–H	3109	C–H	3437	C–H	3239	C–H
3443	C–H	1919	CH_3_	3283	C–H	2945	C–H
2940	C–H	1555	C–C	3167	C–H	1597	C–C
2916	C–H	1397	OH^−^	1649	C–C	1389	OH^−^
1680	C=C	1340	CH_3_	1568	CH_2_	1339	CH_3_
1622	C–C	1288	CH_3_	1410	OH^−^	1287	CH_3_
1478	CH_3_	1242	CH_3_	1024	CH_3_	1146	CH_3_
1402	OH^−^	1003	CH_3_	925	CH_3_	1053	CH_3_
1321	CH_3_	974	CH_3_	891	CH_3_	1026	CH_3_
1254	CH_3_	886	CH_2_	852	CH_2_	936	CH_3_
1180	CH_3_	783	C–H	804	CH_2_	829	CH_2_
1134	CH_3_	702	C–H	-	-	756	C–H
1057	CH_3_	638	C–H	-	-	687	C–H
939	CH_3_	569	Zn–O	-	-	615	C–H
885	CH_2_	517	CH_2_, CH_3_	-	-	558	Zn–O
685	C–H	-	-	-	-	-	-
661	C–H	-	-	-	-	-	-
561	CH_2_, CH_3_	-	-	-	-	-	-
520	CH_2_, CH_3_	-	-	-	-	-	-
**Amylopectin**		**ZnO-amylopectin**		-	-	-	-
3233	C–H	3404	C–H	-	-	-	-
2909	C–H	2874	C–H	-	-	-	-
1624	C–C	2800	C–H	-	-	-	-
1412	OH^−^	1554	CH_2_	-	-	-	-
1331	CH_3_	1383	OH^−^	-	-	-	-
1215	CH_3_	1292	CH_3_	-	-	-	-
1117	CH_3_	1136	CH_3_	-	-	-	-
1049	CH_3_	1096	CH_3_	-	-	-	-
1013	CH_3_	978	CH_3_	-	-	-	-
978	CH_3_	886	CH_2_	-	-	-	-
918	CH_3_	785	C–H				
849	CH_2_	740	C–H	-	-	-	-
716	C–H	625	C–H	-	-	-	-
661	C–H	565	Zn–O	-	-	-	-
569	CH_2_, CH_3_	-	-	-	-	-	-
513	CH_2_, CH_3_	-	-	-	-	-	-
417	CH_2_, CH_3_	-	-	-	-	-	-

**Table 4:** Levels of variation of the main variable parameters.

Name of Parameters	Parameter Designation	Levels of Variable Variation			
C((CH_3_COO)_2_Zn), M	x_1_	0.1	0.2	0.3	0.4
pH	x_2_	7	7.5	8	8.5
τ, minutes	x_3_	5	10	15	20
t, °C	x_4_	20	30	40	50

**Table 5:** Experiment planning matrix.

No.	Parameters	No.	Parameters	No.	Parameters
1	a_1_b_1_c_1_d_1_	7	a_2_b_3_c_4_d_1_	13	a_4_b_1_c_4_d_2_
2	a_1_b_2_c_2_d_2_	8	a_2_b_4_c_3_d_2_	14	a_4_b_2_c_3_d_1_
3	a_1_b_3_c_3_d_3_	9	a_3_b_1_c_3_d_4_	15	a_4_b_3_c_2_d_4_
4	a_1_b_4_c_4_d_4_	10	a_3_b_2_c_4_d_3_	16	a_4_b_4_c_1_d_3_
5	a_2_b_1_c_2_d_3_	11	a_3_b_3_c_1_d_2_		
6	a_2_b_2_c_1_d_4_	12	a_3_b_4_c_2_d_1_		

## Data Availability

All data are available upon request from the corresponding author.
